# A Rare Case of Parathyroid Carcinoma Initially Misdiagnosed as a Parathyroid Adenoma

**DOI:** 10.7759/cureus.98794

**Published:** 2025-12-09

**Authors:** Jawad A Khan, Naveed Ahmad

**Affiliations:** 1 Acute Medicine, Sandwell and West Birmingham Hospitals NHS Trust, Birmingham, GBR; 2 Endocrinology and Diabetes, Sandwell and West Birmingham Hospitals NHS Trust, Birmingham, GBR

**Keywords:** case report, endocrine neoplasms, hypercalcemia, immunohistochemistry, parafibromin, parathyroid adenoma, parathyroid carcinoma, primary hyperparathyroidism, surgical excision

## Abstract

Parathyroid carcinoma is an uncommon endocrine malignancy that can resemble a benign parathyroid adenoma, typically presenting with hypercalcaemia and nonspecific symptoms that make preoperative diagnosis difficult. We report the case of a 38-year-old woman from Birmingham, United Kingdom, who presented with recurrent hypercalcaemia associated with body aches, lethargy, polydipsia, and voice changes. Initial laboratory tests revealed elevated calcium and elevated parathyroid hormone levels. Imaging studies identified a small left parathyroid lesion consistent with a functioning adenoma. The patient underwent left superior parathyroidectomy, with postoperative normalization of calcium and parathyroid hormone levels. Histopathological examination unexpectedly revealed parathyroid carcinoma, confirmed by parafibromin immunohistochemistry and review by a second pathologist. Postoperative imaging showed no evidence of metastasis, and the patient remains asymptomatic with normal biochemical values during follow-up. This case highlights the diagnostic challenge of distinguishing parathyroid carcinoma from adenoma and emphasizes the importance of histopathological evaluation and multidisciplinary management.

## Introduction

Parathyroid carcinoma is exceptionally rare, accounting for less than 1% of cases of primary hyperparathyroidism [1,2]. It can closely resemble benign parathyroid adenoma, making preoperative diagnosis particularly challenging [1-3]. Most patients with primary hyperparathyroidism have benign adenomas, but carcinoma should be suspected in cases with markedly elevated calcium levels, a large parathyroid mass, or evidence of local invasion [1-3]. Preoperative differentiation between adenoma and carcinoma is difficult, as imaging and biochemical features often overlap [3]. Early recognition and complete surgical excision are crucial to prevent recurrence and metastatic spread [1,2].

We present a rare case of parathyroid carcinoma in a 38-year-old woman who was initially diagnosed with a benign parathyroid adenoma. This case underscores the importance of thorough histopathological evaluation and multidisciplinary discussion in the management of parathyroid lesions [4].

## Case presentation

A 38-year-old woman with no significant past medical history was referred to the endocrine clinic for evaluation of recurrent hypercalcaemia detected during multiple hospital admissions. She reported generalized body aches, polydipsia, lethargy, and a recent onset of voice changes. There was no history of nephrolithiasis, fractures, or neuropsychiatric symptoms. Her father had a history of thyroid carcinoma. Given this family history, evaluation for multiple endocrine neoplasia (MEN) syndromes was undertaken. Biochemical screening, including plasma metanephrines to exclude pheochromocytoma, was normal. There were no clinical features or laboratory findings suggestive of MEN1 or MEN2, and the patient had no other endocrine tumors. She was a non-smoker and reported occasional alcohol consumption.

On examination, a smooth, firm, non-tender 1 cm lump was palpable in the anterior neck, separate from the thyroid gland and not tethered to underlying structures. Cardiovascular, respiratory, and abdominal examinations were unremarkable. Laboratory investigations over the patient’s clinical course are summarized in Table 1. The labs demonstrate persistent hypercalcaemia and elevated parathyroid hormone (PTH) preoperatively, with normalization of both parameters following surgery.

**Table 1:** Chronological laboratory parameters. PTH: parathyroid hormone

Date	Calcium (mmol/L)	PTH (pmol/L)	Phosphate (mmol/L)	Vitamin D (nmol/L)	Creatinine (µmol/L)	Notes
November 27, 2023	2.84	13	0.79	27	68	Initial presentation: elevated calcium and PTH
Pre-op March 2025	2.95	14	0.76	30	70	Preoperative evaluation: hypercalcaemia persisted
Post-op April 2025	2.40	5	1.1	50	70	Post-left superior parathyroidectomy: calcium and PTH normalized
Follow-up June 2025	2.42	5.2	1.0	52	72	Routine follow-up: stable labs

**Figure 1:**
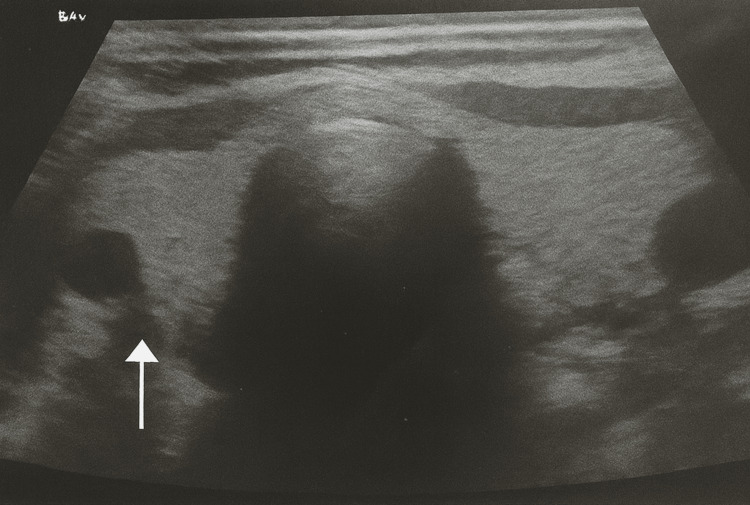
Neck ultrasound showing a 7-mm hypoechoic lesion posterior to the left thyroid lobe (white arrow), consistent with a parathyroid lesion.

A neck ultrasound demonstrated a 7-mm hypoechoic lesion posterior to the left thyroid lobe, consistent with a parathyroid lesion (Figure 1).

**Figure 2:**
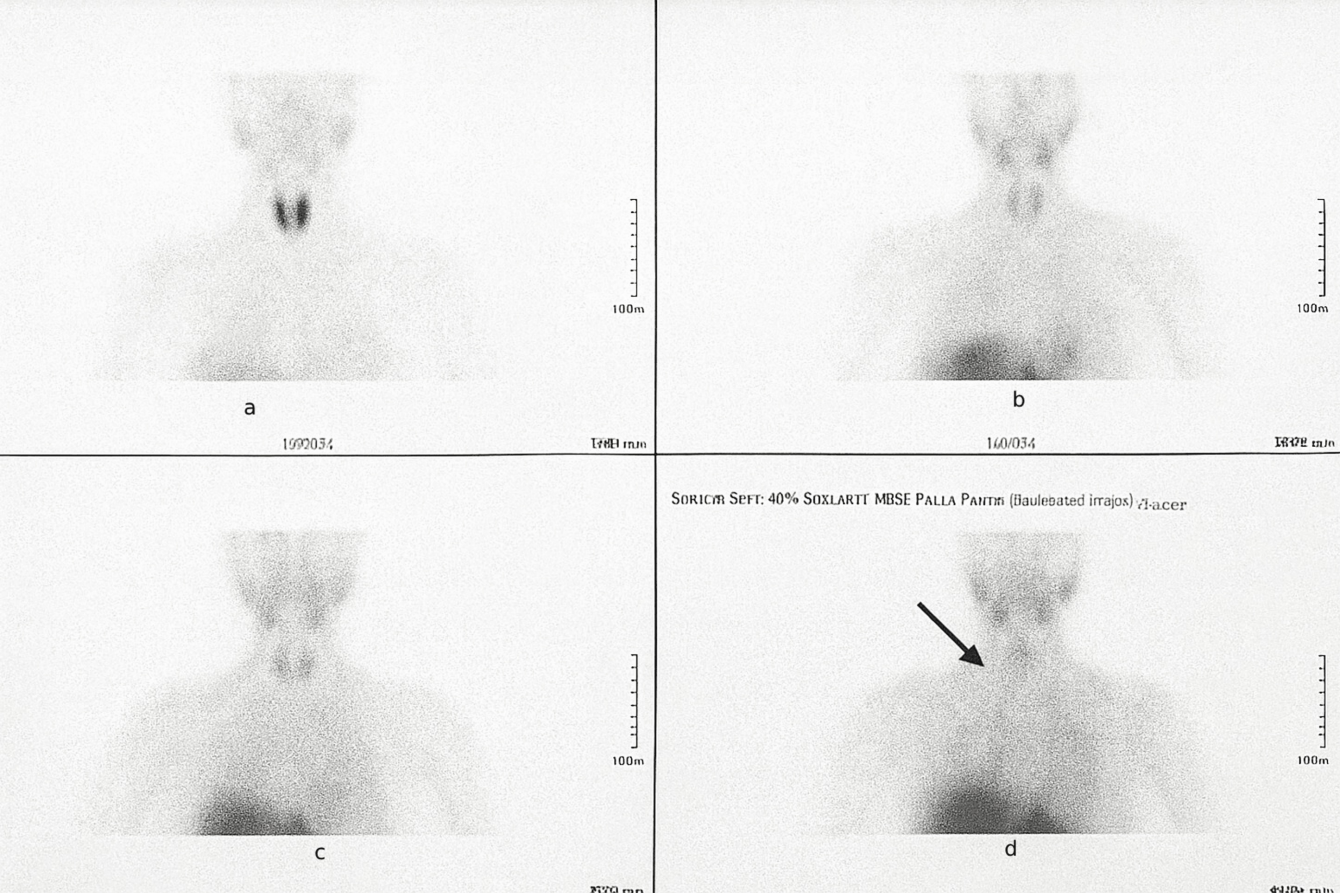
99mTc-sestamibi (MIBI) SPECT scan demonstrating focal tracer uptake corresponding to a 5 × 6 mm nodule posterior to the left thyroid lobe. (a) Early-phase anterior image showing mild asymmetric uptake. (b) Delayed-phase anterior image demonstrating persistent focal tracer retention. (c) Axial/oblique projection showing discrete radiotracer activity posterior to the left thyroid lobe. (d) An annotated image highlighting the lesion (arrow), consistent with a functioning parathyroid adenoma. The lesion is located posterior to the left thyroid lobe as indicated by the arrow. SPECT: single-photon emission computed tomography

A technetium-99m sestamibi (99mTc-sestamibi, MIBI) single-photon emission computed tomography (SPECT) scan demonstrated focal tracer uptake corresponding to a 5 × 6 mm nodule posterior to the left thyroid lobe, suggestive of a functioning parathyroid adenoma (Figure 2).

On March 16, 2025, she underwent a left superior parathyroidectomy. Postoperatively, serum calcium and PTH levels normalized, and she remained biochemically stable. A follow-up neck ultrasound on June 6, 2025, showed no residual or recurrent disease. Unexpectedly, histopathological examination revealed features consistent with parathyroid carcinoma, including capsular and vascular invasion. The diagnosis was confirmed by a second pathologist, and parafibromin immunohistochemistry demonstrated loss of nuclear staining, supporting a malignant diagnosis.

A postoperative CT scan of the neck and thorax (April 29, 2025) showed no evidence of metastasis or lymphadenopathy. The patient remains asymptomatic and under regular endocrine and ENT follow-up with persistently normal calcium and PTH levels.

## Discussion

Parathyroid carcinoma is an exceptionally rare endocrine malignancy, accounting for less than 1% of cases of primary hyperparathyroidism [1,2]. Its clinical presentation often overlaps with benign parathyroid adenoma, making preoperative differentiation difficult. Patients typically present with symptomatic hypercalcaemia, including fatigue, muscle weakness, bone pain, nephrolithiasis, and neurocognitive changes. However, biochemical parameters such as markedly elevated calcium (>3.5 mmol/L) or PTH levels may raise suspicion for carcinoma, although overlap with adenoma frequently occurs. Although the patient had a family history of thyroid carcinoma, evaluation for MEN syndromes was negative, supporting that this parathyroid carcinoma was sporadic rather than syndromic.

Radiological modalities, including neck ultrasound, 99mTc-sestamibi SPECT, and CT scans, are useful for localization but cannot reliably distinguish adenoma from carcinoma. Preoperative or intraoperative ultrasound, including Doppler assessment, may provide additional information on lesion vascularity and characteristics; however, its ability to definitively differentiate benign from malignant parathyroid lesions is limited. In this case, the small lesion size and lack of invasive features on imaging led to the presumptive diagnosis of a benign adenoma, highlighting the inherent limitations of imaging alone in parathyroid pathology.

Histopathological examination remains the gold standard for diagnosis. Features suggestive of carcinoma include dense fibrous trabeculae, capsular and vascular invasion, and increased mitotic activity. Immunohistochemical staining for parafibromin, a tumour suppressor protein encoded by the HRPT2 (CDC73) gene, has emerged as a valuable diagnostic adjunct. Loss of parafibromin expression is highly specific for parathyroid carcinoma and supports the diagnosis, as demonstrated in this case.

The mainstay of treatment is complete surgical excision with negative margins at the initial operation, as reoperation is associated with poorer outcomes. Adjuvant radiotherapy and chemotherapy have shown limited efficacy, and management of recurrent or metastatic disease remains challenging. Long-term follow-up is essential, as recurrence or metastasis may occur years after initial surgery. This includes regular monitoring of serum calcium and PTH levels, with periodic imaging as clinically indicated.

This case underscores the diagnostic difficulty in distinguishing parathyroid carcinoma from adenoma, especially when biochemical and imaging findings are inconclusive. It reinforces the importance of meticulous histopathological evaluation, the use of parafibromin immunohistochemistry, and multidisciplinary management in ensuring accurate diagnosis and optimal patient outcomes.

## Conclusions

Parathyroid carcinoma is a rare and often unexpected cause of primary hyperparathyroidism that can mimic benign adenomas both clinically and biochemically. This case highlights that even small, apparently benign parathyroid lesions may harbour malignancy, emphasizing the critical role of thorough histopathological evaluation and parafibromin immunohistochemistry. Early recognition, complete surgical excision, and long-term multidisciplinary follow-up are essential to optimize patient outcomes and detect potential recurrence.

## References

[1] Schulte KM, Talat N (2012). Diagnosis and management of parathyroid cancer. Nat Rev Endocrinol.

[2] Cetani F, Pardi E, Marcocci C (2019). Parathyroid carcinoma. Front Horm Res.

[3] Shane E (2001). Clinical review 122: parathyroid carcinoma. J Clin Endocrinol Metab.

[4] DeLellis RA (2005). Parathyroid carcinoma: an overview. Adv Anat Pathol.

